# Gender and Generation: Landownership and Older Indians’ Autonomy

**DOI:** 10.1080/13545701.2023.2255878

**Published:** 2023-10-17

**Authors:** Hope Xu Yan, Sonalde Desai, Debasis Barik

**Affiliations:** University of Maryland at College Park - Department of Sociology, 2112 Parren Mitchell Art-Sociology Building (Bldg 146), 3834 Campus Dr., College Park, MD 20742-5031, USA; University of Maryland at College Park - Department of Sociology, College Park, MD, USA; National Council of Applied Economic Research, New Delhi, Delhi, India

**Keywords:** Landownership, gender inequality, older Indians, decision-making power, mortality, J16, J14, Q15

## Abstract

While increased access to household assets has been shown to improve older individuals’ autonomy and bargaining power at home, the role of gender hierarchy in shaping differential impacts of household assets has received far less attention. This article explores the gender asymmetry in the association of older people’s (age 60 years or more) decision-making power at home and survival probability with their ownership of and managerial control over agricultural land in rural India. Using data from the India Human Development Survey, results find that in multi-generational households, landownership at the household level is associated with higher decision-making power and survival probability for older men but not for older women. Among older women, the relationship between household landownership and decision-making power is positive when they have clearly established titles to the land or managerial control but negative when their names are not on the land title.

## INTRODUCTION

In the absence of strong social security systems, research from developing countries in Asia shows that asset ownership is an important source of old age security (Lillard and Willis 1997; Zimmer and Kwong 2003; Lee and Mason 2012; Ladusingh and Maharana 2018). Controlling durable assets such as agricultural land can not only provide economic resources that benefit older individuals but also bring older people more say in household decisions, more access to household resources, more support from family members, and better well-being (Dharmalingam 1994; Amin 1998; Pal 2004; Sudha, Rajan, and Sarma 2004; Takagi and Silverstein 2011; Joshi, Khatiwada, and Chalise 2018). However, little attention has been paid to how gender and generational inequalities in multi-generational households may complicate the relationship between older adults’ ownership of assets and their conversion into intrahousehold power.

Ownership of agricultural land in rural India offers an ideal entry point for examining this question. Much of the agricultural land in India is inherited over generations. Hence, in households with land, age confers power through ownership of a highly valuable resource. However, in rural India, older men usually control household land automatically. Older women, in contrast, are significantly less likely to own household land and may not have effective control over land disposition even when their names are on the land title (Agarwal 1994a, 1994b, 1997, 1998). This gender inequality may moderate the generational power of older women.

In this article, we ask the following questions: (1) Does older women’s autonomy benefit from households’ agricultural land in the same way as older men do? (2) Is it households’ ownership of land or individuals’ control over it that matters most for the autonomy of older men and older women? Using data from the India Human Development Survey (IHDS), we explore the gender asymmetry in how older people’s decision-making power at home varies according to their ownership of and managerial control over households’ agricultural land. Specifically, we focus on variations based on (a) ownership of land at the household level, (b) individuals having their names on the land title, (c) individuals having sole ownership of the household land, and (d) individuals having managerial control over farm-related decision making. To address potential errors in the measure of older people’s decision-making power at home, we also undertake robustness checks using older people’s mortality risk.

## BACKGROUND

### Asset ownership and generational bargains

Asset ownership is an important source of old age security. Unlike younger adults, older people usually do not have labor income as the major source of income. They instead rely largely on asset income, family support, and public financial transfers, where available, for old age support (Torrey and Teauber 1986; Crystal and Shea 1990; Robert and House 1996; Disney, Johnson, and Stears 1998; Hansen, Slagsvold, and Moum 2008). In many Asian developing countries, where public transfers are limited, asset income and family support are particularly important for older people’s economic security and well-being (Lillard and Willis 1997; Zimmer and Kwong 2003; Lee and Mason 2012; Ladusingh and Maharana 2018).

Empirical studies in multiple Asian countries document that owning inheritable assets can increase older people’s bargaining power at home and motivate family members to provide old age support to the older members. For example, older people with higher asset ownership are more likely to receive respect and support from adult children and other family members. Also, it is older people’s economic power instead of their need for care that mainly determines the level of family support (Amin 1998; Pal 2004; Sudha, Rajan, and Sarma 2004; Kodoth and Rajan 2008). Landowners themselves also expect more support from children than their landless counterparts (Dharmalingam 1994). Moreover, older individuals who own the house in which they reside are far more likely to be household heads and be consulted in family decisions than those who live in houses owned by their children or other relatives (Williams and Domingo 1993; Williams, Mehta, and Lin 1999; Takagi and Silverstein 2011). Besides bargaining power at home, studies have also shown that owning key assets such as a house and agricultural land is associated with better health and healthcare utilization (K. Roy and Chaudhuri 2008; Guo, Zhang, and Sherraden 2009; Barik, Desai, and Vanneman 2018), and a higher level of life satisfaction (Han and Hong 2011; Joshi, Khatiwada, and Chalise 2018) among older adults. However, despite the extensive evidence that asset ownership can benefit older people’s autonomy and well-being, little is known about whether older men and older women benefit equally from owning assets such as agricultural land.

### Gendered and generational inequalities in access to and control over land

Agricultural land in India is not easy to sell or transfer, making it mostly an inherited asset buttressed by laws (Morris and Pandey 2007). Over 90 percent of the landowning households in the second wave of IHDS (IHDS-II) acquire the land through inheritance or hold it as undivided family land. Because the inheritance of households’ land by the children’s generation depends on the cooperation of the parental generation, the older generation retains disproportional control over the land. Among land-owning rural households in the IHDS-II sample, about 77 percent of older men (age 60 years or more) and 15 percent of older women were either joint or sole owners of household land. Whereas, for adult men and women under age 60, the corresponding figures were about 22 and 3 percent, respectively.

What can also be seen from the data above is that agricultural land in India is typically controlled by the patriarch, passing down across generations from father to son on the death of the patriarch and sometimes held under joint ownership of the corporate family. Although legislations in 1956 and 2005 have partially established daughters’ rights to inherit fathers’ land and widows’ rights to land owned by their husbands, in practice, few Indian women own agricultural land and even fewer have effective control over it (Agarwal, Anthwal, and Mahesh 2021). This is because, first, granting women legal rights to inherit and own land does not guarantee their actual landownership when the law is not enforced or not considered legitimate by local society and family members. Second, women may only have a claim over the land via or jointly with men family members instead of sole landownership. For example, families may keep or add women members’ names on the land title only because state government policies have encouraged women’s land rights, or they receive the government-distributed land with all family members holding a joint title. In cases like this, older women’s access to the land may only be nominal. They may not have priority over other family members in terms of using and controlling the land and can easily lose their share in the event of a family partition. Third, even with sole ownership, women may still be restricted from deciding how to use the land when there are adult men in the household (Agarwal 1994a, 1994b, 1998).

The gender inequality in landownership suggests that the generational power conferred on older men with landownership may not apply to older women to the same degree, particularly if older women’s control over land is not formally codified or operationally established. It is, therefore, necessary to explore the extent to which gender interacts with control over land to empower older individuals, also to conduct comparisons between older women’s landownership and control over land vis-à-vis nominal access to land. In sum, although prior research has identified gender asymmetry in landownership and control over land, the implication of this gender asymmetry for older individuals’ power at home remains under-studied. This is the area we seek to address in this article.

### Landownership and women’s empowerment

The issue of whether women should exercise their land rights has been the subject of lively debate among feminist economists (Agarwal 2003a, 2003b; Jackson 2003, 2004) because women’s attempts to exercise their rights to agricultural land may lead to family strife and, hence, serve to disempower instead of empower women. A handful of studies have examined how women’s asset ownership shapes intrahousehold power dynamics and women’s autonomy, primarily vis-à-vis their spouses. They generally find women’s land rights and ownership are associated with an increase in their mobility and participation in household decision making (Fafchamps and Quisumbing 2002; Panda and Agarwal 2005; Doss 2006; Allendorf 2007; S. Roy 2008; Deere and Twyman 2012; Oduro, Boakye-Yiadom, and Baah-Boateng 2012; Swaminathan, Lahoti, and Suchitra 2012; Wiig 2013; Doss et al. 2014; Mishra and Sam 2016). However, these studies usually rely on adult samples and combine women of all ages. Consequently, the interaction of gender and generation and the experience of older women have received little attention. Given older individuals’ higher control over household land and their heavy reliance on asset ownership for old age security, as discussed above, the consequence of landownership for older women’s autonomy and empowerment requires special attention.

Further, due to data limitations, many current studies on landownership and women’s empowerment use only women’s legal rights to land inheritance instead of their actual landownership or control over land to estimate the impact of landownership (S. Roy 2008; Wiig 2013). Even when using individual land or asset ownership data, they either do not differentiate between joint title, sole ownership, and actual control over the asset (Panda and Agarwal 2005; Doss 2006; Allendorf 2007; Mishra and Sam 2016) or focus only on the share of ownership between married couples (Deere and Twyman 2012; Oduro, Boakye-Yiadom, and Baah-Boateng 2012; Swaminathan, Lahoti, and Suchitra 2012; Jacobs and Kes 2015; Widman and Hart 2019).

### The present study

The IHDS data offer a unique opportunity to distinguish between households’ ownership of land, individuals’ joint or sole ownership of household land secured by a registered title, and control of land in terms of making decisions about agricultural operations. We link these different dimensions of access to and control over land to rural older Indians’ (age 60 years and above) power over household decisions, which is a key indicator of individuals’ autonomy and control over the distribution of household resources (Manser and Brown 1980; Friedberg and Webb 2006; Doss 2013). We ask the following questions:

1. How is older people’s decision-making power at home associated with their ownership of agricultural land?
2. Does this relationship vary by the nature of ownership and operational control? We are particularly interested in variations based on:
  - Ownership of land at the household level,
  - Individuals having their names on the land title,
  - Individuals having sole ownership of the household land, and
  - Individuals having managerial control over farm-related decision making.
3. Are these relationships heterogeneous across genders? If so, what are the patterns of the variation? For example, is household ownership of land sufficient to confer a greater degree of power on older women as it is on older men? Alternatively, do older women need to own and control the land themselves to have better outcomes?

Data on household decision making in the IHDS were reported by a woman household member of reproductive age, implying that we may measure older people’s decision-making power with some imprecision. To address potential measurement errors in responses to questions about decision-making power, we undertake robustness checks using data on older people’s probability of dying between the first and second waves of IHDS. Past research has documented close relationships between older people’s asset ownership, bargaining power at home, and well-being and mortality risk. As mentioned above, owning key household assets is associated with better health and healthcare utilization among older adults in Asian developing countries (K. Roy and Chaudhuri 2008; Guo, Zhang, and Sherraden 2009; Barik, Desai, and Vanneman 2018). The positive relationships between women’s bargaining power at home and their health, healthcare utilization, and survival probability are also well-documented in the Global South (K. Roy and Chaudhuri 2008; Osamor and Grady 2016; Calvi 2020).

## DATA AND METHODS

### Data

We drew upon data from the first (2004–05) and second (2011–12) waves of the India Human Development Survey, a nationwide longitudinal survey of over 40,000 households. We used the IHDS-II data to study the relationship between older people’s landownership and decision-making power at home. IHDS-II contains 9,233 older individuals age 60 years or older who lived in rural households that had a reproductive-age woman to answer the household decision-making questions. We restricted the sample of analysis to older people living in multi-generational households (*N* = 9,029) given our interests in the role of both gender and generation in intrahousehold power dynamics but also because about 98 percent of the households that had both older people and a reproductive-age woman were multi-generational. After cases with missing values on one or more variables were dropped, the final sample size of analysis was 8,538 (3,876 older men and 4,662 older women). In view of the low amount of missing data, we resorted to list-wise deletion rather than using multiple imputation techniques.

The relationship between older people’s landownership and mortality was analyzed among the IHDS-I older people with tracking records in the IHDS-II. Among the 9,841 older people who lived in rural multi-generational households in the first wave, 9,547 had a tracking record in the second wave. After dropping cases with missing values, the final sample included 9,313 older people (4,356 men and 4,957 women). We also reported results that included older people who did not live in multi-generational households in the sample of analysis (5,940 older men and 5,912 older women) for comparison purposes in Appendix Table A4.

### Measures

#### Dependent variables: Older people’s decision-making power at home and mortality

We measured older people’s decision-making power at home by counting the number of household decisions they had a final say on. A woman of reproductive age (an ever-married woman in the age group of 15–49 years) in the household was asked to identify the primary decision maker for each of the following decisions: (1) what to cook on a daily basis; (2) whether to buy an expensive item, such as a TV or a refrigerator; (3) how many children they should have; (4) what to do if they fall sick; (5) whether to buy land or property; (6) how much money to spend on a social function such as a marriage (Cronbach’s alpha = 0.76). Decisions (7) what to do if a child falls sick and (8) to whom your children should marry were not included in the measure because they were only asked to respondents with children. Decision (1) what to cook on a daily basis may not necessarily reflect older men’s and older women’s decision-making power because cooking is typically in women’s domain. Older men may pay little attention to it regardless of their power of decision at home. Whereas, older women with low bargaining power may still need to decide what to cook. Including decision (1) in the measure of decision-making power or not had little impact on the results of this study. We present results that included decision (1).

The woman of reproductive age (respondent) did not specify which family member was the primary decision maker but only selected from the following options: “respondent,” “husband,” “senior male,” “senior female,” and “others.” We first recoded “husband” into “senior male” if the respondent’s husband was 60 or older and then counted the number of decisions that the “senior male” and “senior female” had a final say on, respectively. Because the number of senior people, especially senior women who had a final say on five or six household decisions was small, we combined the two categories. Depending on the respondent’s age, about 23 percent of the sample households had more than one “senior male” older than the respondent and about 10 percent had more than one “senior female.” The “senior” members that the respondents in these households referred to may, therefore, not be the older men or women we focused on. To solve this problem, we tested if the results still hold after dropping households with more than one “senior male/female” from the sample of analysis. The results remained robust when using this sub-sample (2,738 older men and 4,035 older women). We present the results of the full sample analysis. The mortality risk of older people was measured by whether older individuals interviewed in the IHDS-I had passed away in the IHDS-II.

#### Independent variables: Older people’s landownership status and managerial control over household farm-related decisions

In addition to the questions “does the household own any agricultural land?” and “who is the primary decision maker about farm matters in your household (list only one member)” in both waves of the IHDS, the second wave of IHDS also asked “the land is in the name of which household member (list at most three members)?” Because less than 3 percent of the land-owning rural households listed three landowners, the chance of household members being joint landowners but not listed was extremely low. Based on respondents’ answers to these questions, we measured older people’s landownership and control over land using the following dummy variables: (1) whether the household owns agricultural land, (2) whether the older individual’s name is listed on the household land title, (3) whether the older individual is the sole owner of the household land with no other household members listed as landowners, and (4) whether the older individual is the primary decision maker of household farm matters (0 = No; 1 = Yes). Older people who were primary decision makers of farm matters were more likely to be joint or sole owners of household land and worked more actively in household farms than those who were not (Appendix Table A1). Therefore, primary decision makers of household farm matters were likely to have more control over household land, but this control might mainly be managerial control over agricultural production. Whether they also had control over the inheritance and trading of the land is unclear.

We also constructed a mutually exclusive categorical landownership variable, coded as 0 = the household does not own land; 1 = the household owns land, but the older individual is not listed as one of the landowners; 2 = the older individual is a joint owner but not the sole owner of the household land; and 3 = the older individual is the sole owner of the household land.

#### Covariates

The other independent variables included older people’s age, a squared term for age, marital status (married and widowed/divorced), years of education, as well as the natural log of households’ annual income, number of assets or consumer durables owned by the household from a select list, size of agricultural land, home ownership (owned and not owned), household size (comprising the number of household adult men and adult women), and the highest education level of household members. We also controlled for household heads’ caste and religion (Forward Caste, OBC, Dalit, Adivasi, Muslim, and Christian/Sikh/Jain) and households’ state of residence because gender norms and women’s rights to inherit land vary by state and religion (Desai and Temsah 2014; Agarwal, Anthwal, and Mahesh 2021).

### Analytic strategies

To measure the gender asymmetry in the association between older people’s landownership status and decision-making power at home, we first estimated a series of ordered logistic regression models with the dependent variables being the number of household decisions that older men and older women had a final say on and the independent variables being each of the four dummy landownership variables. Cross-gender group comparisons were made using Stata’s *suest* and *test* command to check the significance of gender differences. We then estimated ordered logistic regression models with the mutually exclusive categorical landownership variable as the independent variable. To better understand the gender and generational power dynamics in multi-generational households, we also explored how living arrangements moderated the association between older women’s landownership and bargaining power at home. This was done by interacting older women’s landownership status with their marital status, and the number of household adult men and women under age 60, respectively. The parallel regression assumption (PRA), which assumes the coefficient for each independent variable in the separate cumulative logit model to be identical, was not violated by the key landownership variables.

We examined the relationships between landownership status and mortality risk for older men and women using binary logistic regression models. Because the individual landownership data was not available in the first wave, we could only estimate the models that included indicators for whether the household owned land and whether the older individual was the primary decision maker of household farm matters as the key independent variables. All analyses were weighted with standard errors being adjusted for clustering on PSUs.

## RESULTS

### Descriptive statistics

Table 1 and Appendix Table A2 show the demographic characteristics of older men and older women with different landownership conditions in IHDS-II multi-generational households. Among households that owned agricultural land, about 77 percent (2,299 out of 2,975) of the older men were owners of their households’ land, and the majority of them were also sole landowners and primary decision makers of household farm operations. In contrast, less than 15 percent (488 out of 3,377) of the older women had their names on the household land title. Among them, only about half were sole owners of their households’ land. The percentage of older women who made primary decisions for household farm matters was even lower.

In general, households with agricultural land had higher socioeconomic status (in terms of household income, caste, and household members’ education levels) than landless households (Table A2). Among landed households, older men and women who were landowners (either sole or joint) or primary decision makers regarding farm matters were generally younger and received more years of education than those who were not (Table 1). Over 80 percent of the older women who owned the household land jointly and almost 90 percent of those who owned the household land solely or made primary decisions for farm operations were widowed or divorced. This may mean that older women mainly inherited and gained control over household land after the death of their husbands. In contrast, the incidence of landownership for older men was relatively independent of their marital status.

### Decision-making power at home

Compared with older men, older women had lower decision-making power at home, with about 68 percent not having a final say on any of the six household decisions (Table 2). Older men who lived in landed households had more decision-making power over all the six household decisions than those living in landless households (Table 3). In contrast, living in landed households did not necessarily mean a greater say on household decisions for older women. For most household decisions, older women were slightly more likely to have a final say when living in landless households. Among both older men and women, being sole/joint owners of household land or primary decision makers of household farm matters were generally associated with a greater say on household decisions, but the variations in decision-making power by landownership conditions were especially salient among older women.

The results of ordered logistic models in Table 4 are consistent with what we have observed in Table 3.

#### Ownership of land at the household level – Models 1a and 1b

Landownership at the household level was associated with a higher likelihood (OR = 1.35, *p* = 0.005) of having a final say on more household decisions for older men (Model 1a) but not for older women (Model 1b). Older women tended to have lower rather than higher decision-making power when living in land-owning households though this difference was not statistically significant (OR = 0.85, *p* = 0.264). The cross-group comparison results suggest that the gender difference in odds ratios was statistically significant (*p* = 0.008).

#### Individuals having their names on the land title – Models 2a and 2b

Another gender asymmetry was that as long as households owned land, the issue of whether older individuals had their names on the land title did not make any significant difference in older men’s decision-making power (Model 2a). In contrast, for older women, having names on the land title was associated with a higher likelihood of having a final say on more household decisions (OR = 2.53, *p* < 0.001, Model 2b), with the gender difference in odds ratios being statistically significant at the 0.001 level.

#### Individuals having sole ownership of the land – Models 3a and 3b

Similarly, for older men, there was a very small and statistically insignificant positive effect associated with being sole landowners (Model 3a). However, for older women, having their names on the land title without any co-owners was associated with a large and statistically significant (OR = 3.06, *p* < 0.001) improvement in intrahousehold decision-making power (Model 3b).

#### Individuals having control over farm-related decision making – Models 4a and 4b

Both older men and older women, who were primary decision makers regarding household farm matters, were more likely to be primary decision makers in other household decisions (*p* < 0.001 for both older men and older women). However, this association was far stronger for older women than older men – the odds ratio on ordinal logit for men was 2.01 as opposed to 4.67 for women, and this difference was statistically significant (*p* = 0.002).

**Figure 1:**
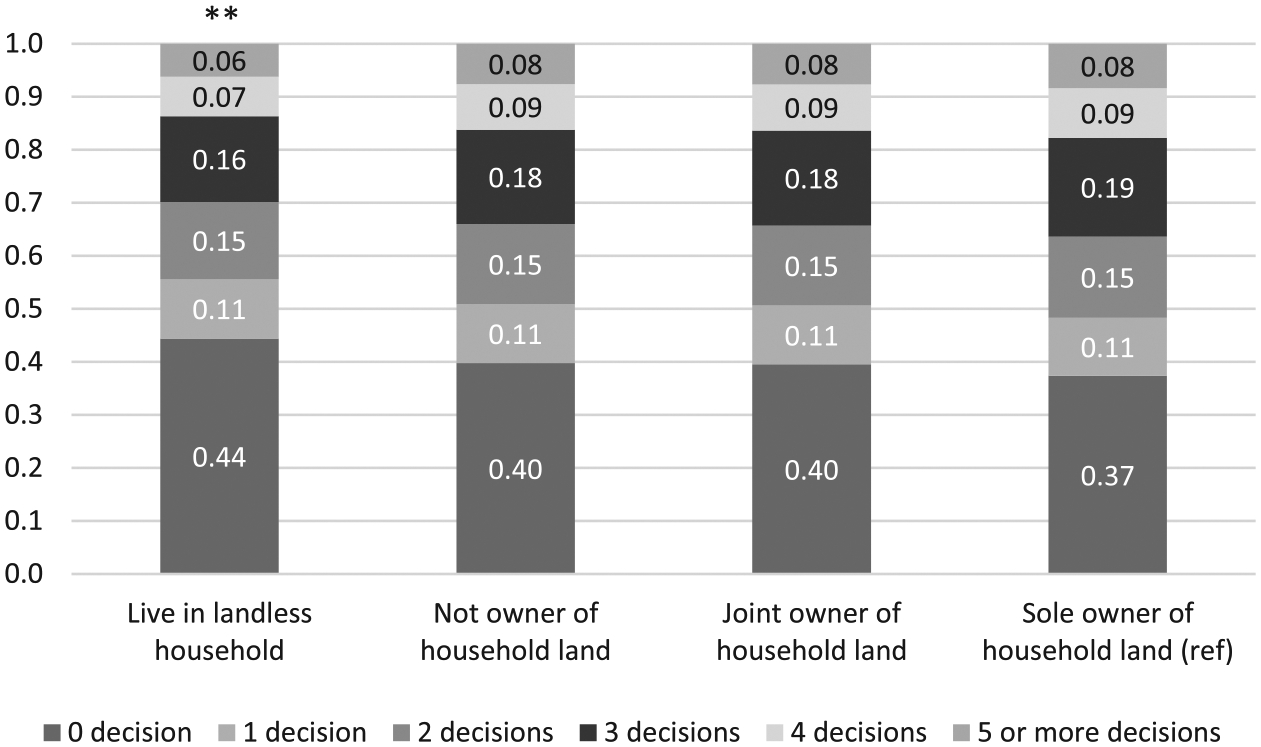
Predicted probabilities for the number of household decisions older men have a final say on by landownership in the IHDS-II (*N* = 3,876). *Notes*: Report predicted probabilities from ordered logit models. Reference group: older men as sole owners of household land. ** denotes statistical significance at the 1 percent level.

We also estimated ordered logistic regression models using the mutually exclusive landownership variable as the independent variable. Consistent with the results observed in Table 4, the incidence of a household owning land was associated with enhanced decision-making power of older men in that household (Figure 1). Also, as long as older men lived in land-owning households, the predicted probabilities for the number of decisions that older men had a final say on varied little by their individual landownership status.

**Figure 2:**
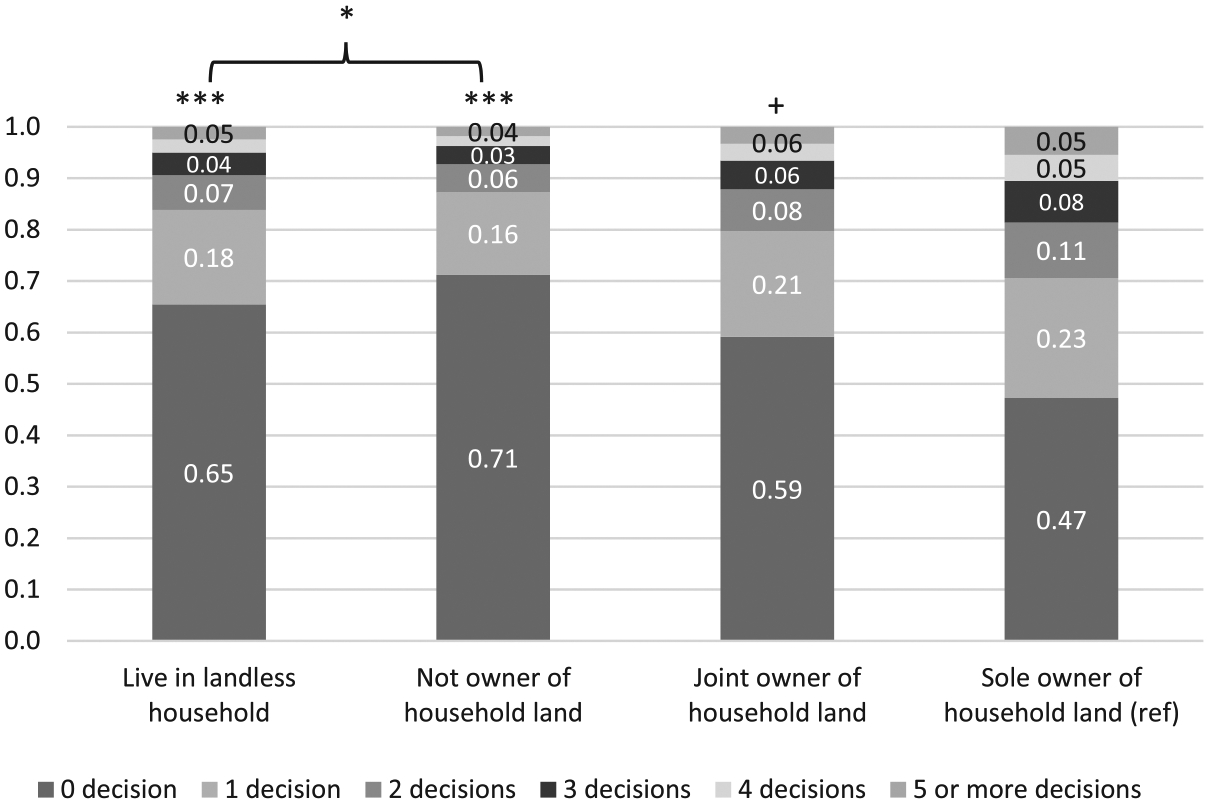
Predicted probabilities for the number of household decisions older women have a final say on by landownership in the IHDS-II (N = 4,662). *Notes*: Report predicted probabilities from ordered logit models. Reference group: older women as sole owners of household land. ***, *, + denote statistical significance at the 0.1, 5, and 10 percent levels, respectively.

In contrast to older men, older women’s decision-making power was much more sensitive to their personal ownership of household land (Figure 2). Older women who lived in landed households without having their names on the land title had significantly lower decision-making power than those residing in landless households (*p* = 0.040). Whereas, among older men, the relationship was reversed (Figure 1), with the gender difference in odds ratios being statistically significant (*p* = 0.009, result not presented). Besides, older women who were sole landowners had a significantly (or marginally significantly) higher likelihood of having a final say on more household decisions than their peers with all other landownership statuses (Figure 2). The cross-gender group comparison results (not presented) also show that the effect size of being sole owners of household land (as compared to living in landless households and not having names on the land title) was significantly larger for older women than for older men.

To examine whether the relationship between older women’s landowner ship and bargaining power at home was sensitive to their living arrangements, we interacted older women’s landownership status with their marital status and the number of household adult men and women under age 60, respectively. Results, as presented in Table 5, show that joint/sole landownership and managerial control over the household farm were associated with greater decision-making power at home only among widowed or divorced older women (Model 1a and Model 1b). Similarly, sole landownership and managerial control over the farm were associated with more bargaining power for older women only when there was one or fewer than one younger man under age 60 in the household (Model 2a and Model 2b). In contrast, the number of household adult women under age 60 had little impact on the relationship between older women’s landownership and decision-making power at home (Model 3a and Model 3b).

### Mortality

Given the potential measurement error in responses to decision-making questions, we conducted robustness checks using older people’s probability of mortality between IHDS-I and IHDS-II. Descriptive statistics (Table 6 and Appendix Table A3) show that older men who lived in landed multi-generational households had a lower mortality risk than their peers in landless households. Whereas, the opposite was true for older women. In landed households, both older men and older women who were primary decision makers regarding household farm matters had a lower risk of mortality than those who were not.

The binary logistic regression results show similar patterns (Table 7). Living in households that owned agricultural land was associated with a 0.23 lower likelihood of dying between two waves of the survey for older men (*p* = 0.036, Model 1a). In contrast, older women residing in landed households faced a slightly higher risk of mortality than those residing in landless households (Model 1b) but the difference was not statistically significant (OR = 1.07, *p* = 0.517). The cross-group comparison result shows the gender difference in odds ratios was statistically significant (*p* = 0.046). Among older men, primary decision makers of household farms had significantly lower odds of mortality than those who were not (OR = 0.48, *p* < 0.001, Model 2a). Among older women, the difference in odds was not significant (OR = 0.71, *p* = 0.204, Model 2b). This lack of statistical significance may be due to the very small sample size of women who were primary decision makers in farm matters (174 women) and the rarity of experiencing mortality. However, given that the gender difference in odds ratios was not statistically significant (*p* = 0.183), the associations between being primary decision makers on household farm matters and mortality risk were similar for older men and women.

When including older people who did not live in multigenerational households in the sample of analysis (Appendix Table A4), households owning land was associated with a 0.17 lower mortality risk for older men, but only at a marginal level of significance (*p* = 0.058, Model 1a), and the gender difference in odds ratios became non-significant (*p* = 0.100). Further, being primary decision makers of household farm matters was associated with a significantly lower mortality risk for both older men (OR = 0.47, *p* < 0.001, Model 2a) and older women (OR = 0.56, *p* = 0.009, Model 2b).

## DISCUSSION

Using the IHDS data, we document an interesting gender asymmetry in the generational power of older people with landownership in rural multi-generational households. Older men hold more power in household decision making when living in landed households than in landless households. With most land in India being inherited through the male line, older men and family patriarchs often control land and have substantial decision-making power at home. Their power appears to be absolute and not contingent on whether their names are listed on the land title or whether they play an important operational role in farm management. In contrast, landownership at the household level is not associated with greater decision-making power at home for older women. Older women have higher levels of decision-making power at home only when their landownership is documented through land titling or operational control of the land. Ironically, older women who live in landed households but do not have their names on the land title appear to be the most disadvantaged. They have even less say on household decisions than those living in landless households. This may be because when older women do not own the household land, their husbands, sons, or other men family members are likely to be the owners. The high decision-making power of the men owners may then restrict older women’s control over household decisions.

Although rare, being the only owner of household land or the primary decision maker regarding household farms is associated with particularly high autonomy at home for older women. Besides their significantly higher decision-making power relative to their peers with other landownership statuses, sole landownership and control over farm-related decisions are also associated with a greater final say on household decisions for older women than for older men. However, this high bargaining power is sensitive to the presence of other family members. Our results suggest that as long as older women’s husbands are alive, husbands retain the power in generational exchanges, and the power of landownership and control accrue to them rather than their wives. Once older women are widowed, those who retain landownership in their own names or are effectively managing farms have substantial bargaining power at home. Whereas, for those living in landless households, not having household land registered under their names, or not managing the household farm, the balance of power may favor the younger generation. Further, sole landownership and managerial control over land are associated with greater bargaining power for older women regardless of the number of younger women under age 60 (likely their daughters and daughters-in-law) in their households. These positive relationships no longer exist when older women co-reside with more than one younger man under age 60 (for example, co-residing with multiple sons).

Our findings remain robust when using older people’s mortality risk as the outcome variable. The incidence of households owning land is associated with a lower mortality risk for older men. In contrast, older women tend to have a slightly higher mortality rate when residing in households that own land. Yet, both older men and women who are primary decision makers regarding household farms have lower mortality risks than those who are not. Due to data limitations, we cannot test the extent to which older people’s survival probability varies according to their landownership conditions at the individual level. Nevertheless, given the gender asymmetric pattern observed in current results, we do expect that older women’s mortality rate to be more sensitive to individuals’ landownership status than that of older men. Older women may even suffer from households’ agricultural land in their survival probability when they do not own or control the land.

The existing literature, as discussed above, has shown that owning key household assets is associated with more bargaining power at home, more respect and support from family members, and better well-being for older people. However, the results presented in this article show the existence of gender asymmetry in the relationship. Households owning inheritable assets such as agricultural land is associated with greater power and respect afforded to older men but not older women. The entrenched gender inequality limits older women’s ability to crystalize this power. They even have lower bargaining power, likely also lower survival probability, when they do not personally own or have managerial control over household land (as compared to living in landless households).

The women’s movement in India has lobbied extensively to ensure that wives and daughters are considered at par with husbands and sons in land inheritance, and women’s rights to land should be recognized by Indian laws (Agarwal 1994a, 1998). Our results suggest that only granting older women legal rights to land is not sufficient to increase their bargaining power at home. At a minimum, the formal recognition and excise of this power by registering the land under women’s names is required. Older women taking managerial control over household agricultural operations is also associated with greater intrahousehold bargaining power and lower mortality. Yet, older women who attain managerial control over household farms are generally younger and participate more actively in household agricultural production than those who do not, making it questionable to what extent their managerial control over land, as well as their high bargaining power and low mortality risk, would persist as they age. Further, in multi-generational households, older women’s ownership to and managerial control over household land may no longer be associated with greater bargaining power at home when they coreside with husbands and/or multiple sons. Therefore, having family members, particularly men members, recognize women as actual instead of nominal landowners is also critical.

While our study is conducted using data from India, women’s lack of control over household assets and low bargaining power at home are widely observed in developing countries (Deere, Alvarado, and Twyman 2012; Doss 2013). The gender asymmetry in the relationship between older Indian’s landownership and autonomy, as well as older women’s disadvantages in bargaining power at home when not owning or having managerial control over household land may, therefore, also exist in other developing countries. In the absence of a strong social security system, household assets are important sources of old age security in developing countries (Lillard and Willis 1997; Zimmer and Kwong 2003; Lee and Mason 2012; Ladusingh and Maharana 2018), making it crucial to explore strategies that can effectively increase women’s ownership and control over household land in different national contexts.

It is important to note that much of our analysis suggests but cannot establish that the relationship between older people’s landownership and intrahousehold power is causal. Older individuals may own or control household land because they have more decision-making power at home and better health, instead of the other way around. While this endogeneity may not limit our ability to draw conclusions about the existence of gender asymmetry, we argue with caution that registering household land under older women’s names and letting them control agricultural operations have the potential for reshaping household power dynamics and improving older women’s bargaining power at home and survival probability.

Another limitation of this study is that older people’s mortality risk is only used for robustness checks, and its analysis is restricted by data limitations. Future studies can explore the extent to which landownership can be translated into improvements in health and survival probability for older men and older women. Further, because we focus on the role of both gender and generation in intrahousehold power dynamics and the household decision-making questions were predominantly asked in multi-generational households, we do not include older people who live alone or only with their spouses and/or unmarried children in this analysis. We do find that the gender asymmetry in the association between household landownership and older people’s mortality risk diminishes when including older people who do not live in multi-generational households, suggesting that the power dynamics in single, couple, and nuclear households may be different from that of multi-generational households.

Despite these limitations, this study is among the first to provide empirical evidence on the existence of gender asymmetry in the association between landownership and older people’s autonomy in India. We hope that future research with longitudinal data will help address the potential endogeneity and draw a more accurate causal inference. With the incipient population aging and the lack of well-established social security systems in many developing countries, it is important to ensure that older citizens, particularly older women, can gain care and support from their children and other family members. Access to and control over land may well be an important avenue to ensuring that.

## Figures and Tables

**Table 1:** Weighted means (standard deviations) or percentage distributions for selected covariates by older people’s gender and landownership in the IHDS-II

	Older men		Older women	
	Landed household	Landless household	Landed household	Landless household
Age	69.28 (7.82)	69.40 (8.02)	69.14 (7.99)	69.10 (7.52)
Widowed/divorced (%)	24.1	27.5	57.9	70.6
Years of education	3.72 (4.25)	2.84 (3.71)	0.79 (2.14)	0.94 (2.30)

No. of household adult men	2.31 (0.89)	2.15 (0.76)	1.74 (0.98)	1.45 (0.85)
No. of household adult women	2.12 (0.87)	1.94 (0.77)	2.36 (0.71)	2.25 (0.59)
*N*	2,975	901	3,377	1,285
	Landowner	Not landowner	Landowner	Not landowner
Age	68.72 (7.41)	71.08 (8.51)	68.27 (7.07)	69.28 (8.08)
Widowed/divorced (%)	22.2	30.0	81.6	54.0
Years of education	3.83 (4.30)	3.36 (3.92)	1.27 (2.73)	0.71 (2.00)
No. of household adult men	2.30 (0.88)	2.33 (0.88)	1.52 (0.93)	1.77 (0.98)
No. of household adult women	2.12 (0.86)	2.08 (0.88)	2.34 (0.70)	2.37 (0.71)
*N*	2,299	676	488	2,889
	*Older men*		*Older women*	
	Sole landowner	Not sole landowner	Sole landowner	Not sole landowner
Age	68.53 (7.31)	70.61 (8.32)	68.19 (7.32)	69.21 (8.00)
Widowed/divorced (%)	21.3	29.0	89.4	55.2
Years of education	3.74 (4.30)	3.70 (4.08)	0.77 (2.02)	0.79 (2.13)
No. of household adult men	2.23 (0.83)	2.45 (0.94)	1.34 (0.86)	1.77 (0.98)
No. of household adult women	2.08 (0.81)	2.19 (0.95)	2.29 (0.61)	2.37 (0.71)
*N*	1,918	1,057	277	3,100
	Decision maker	Not decision maker	Decision maker	Not decision maker
Age	67.18 (6.40)	72.78 (8.49)	65.87 (5.86)	69.32 (8.02)
Widowed/divorced (%)	15.0	39.2	89.5	56.1
Years of education	4.24 (4.34)	2.86 (3.86)	0.92 (2.62)	0.78 (2.10)
No. of household adult men	2.25 (0.90)	2.40 (0.84)	1.22 (1.00)	1.77 (0.97)
No. of household adult women	2.21 (0.84)	1.97 (0.88)	2.37 (0.71)	2.34 (0.66)
*N*	1,872	1,103	186	3,191

**Table 2:** Weighted distributions (%) for the number of decisions older men and women have a final say on in the IHDS-II

	0	1	2	3	4	5–6	N
Older men	39.6	10.8	14.8	18.1	9.0	7.8	3,876
Older women	67.5	17.4	6.4	4.2	2.3	2.2	4,662

**Table 3:** Weighted distributions (%) for the decision-making power of older men and women by landownership in the IHDS-II

	What to cook	Whether to purchase a large item	No. of children the younger generation should have	What to do when younger women fall sick	Whether to buy land or property	Spend how much money on social functions
Older men have a final say (%)						
Landed household	10.6	43.9	7.5	20.2	54.1	46.1
Landless household	9.9	32.7	5.3	16.8	41.2	34.7
Landowner	11.6	46.2	8.1	20.7	55.7	48.0
Not landowner	7.3	36.3	5.6	18.4	49.0	39.8
Sole landowner	12.0	46.4	7.9	20.7	56.3	48.7
Not sole landowner	8.2	39.3	6.7	19.2	50.1	41.4
Decision maker	12.7	53.2	8.9	24.7	63.0	55.3
Not decision maker	7.2	28.4	5.2	12.6	39.3	30.6
Older women have a final say (%)						
Landed household	25.1	7.5	3.4	9.2	7.7	10.7
Landless household	20.1	8.7	3.6	8.2	11.1	12.4
Landowner	31.5	16.1	3.8	13.8	15.4	20.6
Not landowner	24.0	6.1	3.3	8.4	6.4	9.1
Sole landowner	30.5	20.3	4.2	12.1	19.7	24.6
Not sole landowner	24.6	6.5	3.3	8.9	6.7	9.6
Decision maker	32.8	27.4	5.4	16.5	25.8	35.3
Not decision maker	24.6	6.4	3.3	8.7	6.6	9.3

**Table 4:** Odds ratios for ordered logit models predicting the number of household decisions older men and women have a final say on by landownership in the IHDS-II

	Household owns land (ref. no)			Landowner (ref. no)			Sole landowner (ref. no)			Decision maker (ref. no)		
	Model 1a Men	Model 1b Women	M vs. W	Model 2a Men	Model 2b Women	M vs. W	Model 3a Men	Model 3b Women	M vs. W	Model 4a Men	Model 4b Women	M vs. W
Yes	1.350** (0.145)	0.852 (0.122)	*p* = 0.008	1.106 (0.134)	2.528*** (0.403)	*p* < 0.001	1.112 (0.116)	3.058*** (0.595)	*p* < 0.001	2.010*** (0.217)	4.669*** (1.273)	*p* = 0.002
Age	0.825* (0.066)	0.796** (0.058)		0.789** (0.067)	0.714*** (0.054)		0.789** (0.067)	0.722*** (0.054)		0.796** (0.067)	0.732*** (0.055)	
Age squared	1.001+ (0.001)	1.001** (0.000)		1.001* (0.001)	1.002*** (0.001)		1.001* (0.001)	1.002*** (0.001)		1.001* (0.001)	1.002*** (0.001)	
Widowed/Divorced (ref. married)	0.733* (0.107)	0.741* (0.103)		0.908 (0.081)	0.824** (0.059)		0.910 (0.080)	0.841* (0.060)		0.948 (0.082)	0.837* (0.059)	
Years of education	1.051*** (0.013)	1.054** (0.020)		1.064*** (0.015)	1.050+ (0.027)		1.064*** (0.015)	1.061* (0.027)		1.059*** (0.015)	1.060* (0.026)	
Household caste and religion (ref. Forward)												
OBC	0.882 (0.112)	0.879 (0.108)		0.891 (0.126)	0.901 (0.128)		0.893 (0.126)	0.915 (0.129)		0.903 (0.130)	0.927 (0.131)	
Dalit	0.903 (0.139)	1.102 (0.195)		0.869 (0.158)	0.936 (0.173)		0.869 (0.158)	0.937 (0.171)		0.875 (0.161)	0.944 (0.173)	
Adivasi	0.940 (0.190)	0.925 (0.195)		0.933 (0.223)	0.752 (0.171)		0.928 (0.222)	0.800 (0.190)		0.923 (0.225)	0.746 (0.171)	
Muslim	1.090 (0.212)	0.735 (0.154)		1.053 (0.252)	0.614+ (0.166)		1.057 (0.253)	0.611+ (0.165)		1.035 (0.250)	0.607+ (0.166)	
Christian/Sikh/Jain	0.526* (0.170)	0.819 (0.182)		0.447 (0.245)	1.091 (0.392)		0.453 (0.249)	1.212 (0.437)		0.412 (0.250)	1.113 (0.417)
Household income (logged)	1.091+ (0.058)	1.030 (0.061)		1.086 (0.062)	1.060 (0.069)		1.087 (0.062)	1.050 (0.071)		1.074 (0.063)	1.043 (0.070)
No. of household assets	1.009 (0.012)	1.045*** (0.014)		1.003 (0.013)	1.022 (0.015)		1.004 (0.013)	1.026+ (0.014)		1.005 (0.013)	1.026+ (0.015)
Land size (hectares)	0.982 (0.023)	0.986 (0.022)		0.981 (0.024)	0.975 (0.024)		0.983 (0.024)	0.991 (0.023)		0.986 (0.024)	0.986 (0.023)
Owned house (ref. not owned)	3.452 (3.949)	0.279** (0.135)		0.325 (0.308)	0.197+ (0.164)		0.325 (0.308)	0.188* (0.154)		0.292 (0.302)	0.077* (0.084)
Household members highest education	0.991 (0.014)	1.011 (0.012)		0.992 (0.016)	1.009 (0.015)		0.992 (0.016)	1.011 (0.015)		0.989 (0.016)	1.009 (0.015)
No. of household adult men	0.926 (0.055)	1.039 (0.080)		0.908 (0.064)	1.016 (0.098)		0.912 (0.064)	1.021 (0.098)		0.950 (0.065)	1.035 (0.099)
No. of household adult women	1.412*** (0.101)	1.443*** (0.121)		1.398*** (0.119)	1.524*** (0.147)		1.399*** (0.119)	1.513*** (0.143)		1.343*** (0.116)	1.500*** (0.140)
Wald (df = 39)	450.65	365.33		332.76	298.99		334.54	305.79		371.13	286.41
*N*	3,876	4,662		2,975	3,377		2,975	3,377		2,975	3,377

*Notes*: Report odds ratios with standard errors in parentheses from ordered logit models. Covariate state of resident was omitted.

***, **, *, + denote statistical significance at the 0.1, 1, 5, and 10 percent levels, respectively.

**Table 5:** Odds ratios for ordered logit models predicting the number of household decisions older women have a final say on by landownership and living arrangements in the IHDS-II

	Model 1a	Model 1b	Model 2a	Model 2b	Model 3a	Model 3b
Landownership (ref. live in landless household)						
Not owner of household land	0.670 (0.167)		0.746 (0.139)		0.735+ (0.132)	
Joint owner of household land	0.352* (0.142)		1.171 (0.363)		1.120 (0.342)	
Sole owner of household land	0.864 (0.548)		3.005*** (0.766)		2.471*** (0.637)	
Decision maker of household farm (ref. no)		0.686 (0.455)		7.033*** (2.082)		5.017*** (1.342)
Widowed/Divorced (ref. married)	0.522* (0.145)	0.659** (0.094)				
Landownership*Widowed/Divorced						
Not owner of household land*Widowed/Divorced	1.133 (0.319)					
Joint owner of household land*Widowed/Divorced	6.595*** (3.095)					
Sole owner of household land*Widowed/Divorced	3.347+ (2.232)					
Decision maker of farm*Widowed/Divorced		8.891** (6.418)				
Household has more than one adult man under 60 (ref. no)			1.314 (0.282)	1.347 (0.188)		
Landownership*Household has more than one adult man under 60						
Not owner of household land*Yes			0.991 (0.242)			
Joint owner of household land*Yes			1.532 (0.784)			
Sole owner of household land*Yes			0.394* (0.185)			
Decision maker of farm* Household has more than one adult man under 60				0.227* (0.132)		
Household has more than one adult woman under 60 (ref. no)					1.857** (0.430)	2.033*** (0.295)
Landownership* Household has more than one adult woman under 60 (ref. no)						
Not owner of household land*Yes					1.025 (0.243)	
Joint owner of household land*Yes					1.829 (0.879)	
Sole owner of household land*Yes					0.777 (0.402)	
Decision maker of farm* Household has more than one adult woman under 60						0.761 (0.557)
Wald (df)	401.58 (44)	299.30 (40)	407.27 (44)	295.29 (40)	394.87 (44)	290.22 (40)
*N*	4,662	3,377	4,662	3,377	4,662	3,377

*Notes*: Report odds ratios with standard errors in parentheses from ordered logit models. Odds ratio for covariates were omitted.

***, **, *, + demote statistical significance at the 0.1, 1, 5, and 10 percent levels, respectively.

**Table 6:** Weighted means (standard deviations) or percentage distributions for older people’s mortality rate between the IHDS-I and IHDS-II and selected covariates by gender and landownership in the IHDS-I

	Older men		Older women	
	Landed household	Landless household	Landed household	Landless household
Mortality (%)	33.5	38.1	31.4	28.1
Age	68.68 (7.99)	67.71 (7.17)	68.11 (8.04)	67.14 (6.87)
Widowed/divorced (%)	24.2	24.2	55.7	65.7
Years of education	3.13 (3.88)	2.23 (3.41)	0.63 (1.89)	0.57 (1.72)
No. of household adult men	2.45 (0.99)	2.20 (0.84)	1.85 (1.07)	1.54 (0.90)
No. of household adult women	2.10 (0.92)	1.78 (0.78)	2.17 (0.91)	1.98 (0.90)
*N*	3,204	1,152	3,490	1,467
	Decision maker	Not decision maker	Decision maker	Not decision maker
Mortality (%)	22.2	44.0	20.6	29.4
Age	66.22 (6.07)	72.39 (8.84)	64.93 (6.57)	68.27 (7.99)
Widowed/divorced (%)	13.2	40.7	90.7	53.6
Years of education	3.62 (4.14)	2.38 (3.32)	1.06 (3.00)	0.61 (1.80)
No. of household adult men	2.35 (0.99)	2.60 (0.95)	1.03 (1.00)	1.90 (1.05)
No. of household adult women	2.33 (0.79)	2.06 (0.57)	2.09 (0.72)	2.35 (0.78)
*N*	1,972	1,220	192	3,280

**Table 7:** Odds ratios for binary logit models predicting older men and women dying between the IHDS-I and IHDS-II by landownership in the IHDS-I

	Household owns land (ref. no)			Decision maker (ref. no)		
	Model 1a Men	Model 1b Women	M vs. W	Model 2a Men	Model 2b Women	M vs. W
Yes	0.772* (0.095)	1.072 (0.116)	*p* = 0.046	0.483*** (0.058)	0.714 (0.189)	*p* = 0.183
Age	1.197 (0.131)	1.103 (0.091)		1.262+ (0.157)	1.208* (0.113)	
Age squared	0.999 (0.001)	1.000 (0.001)		0.999 (0.001)	0.999 (0.001)	
Widowed/divorced (ref. married)	1.350*** (0.086)	1.268*** (0.076)		1.321*** (0.102)	1.254*** (0.083)	
Years of education	0.971+ (0.016)	0.924** (0.026)		0.981 (0.019)	0.920* (0.033)	
Household caste and religion (ref. Forward)						
OBC	0.875 (0.122)	1.117 (0.145)		0.951 (0.154)	1.240 (0.193)	
Dalit	1.008 (0.150)	1.167 (0.181)		1.114 (0.206)	1.118 (0.247)	
Adivasi	1.219 (0.232)	1.868** (0.409)		1.126 (0.257)	1.917* (0.493)	
Muslim	0.922 (0.192)	1.211 (0.239)		1.048 (0.251)	1.102 (0.278)	
Christian/Sikh/Jain	1.283 (0.364)	1.055 (0.248)		1.019 (0.473)	0.730 (0.268)	
Household income (logged)	0.986 (0.053)	0.920 (0.052)		1.018 (0.062)	0.927 (0.064)	
No. of household assets	0.964** (0.013)	0.985 (0.012)		0.968* (0.015)	0.981 (0.014)	
Land size (hectares)	1.000 (0.000)	1.000 (0.000)		1.000 (0.000)	1.000 (0.000)	
Owned house (ref. not owned)	0.737 (0.336)	0.582 (0.228)		0.360 (0.313)	0.489 (0.324)	
No. of household adult men	0.951 (0.062)	0.995 (0.064)		0.855* (0.062)	1.023 (0.074)	
No. of household adult women	1.186* (0.090)	1.089 (0.086)		1.200* (0.101)	0.947 (0.085)	
Wald (df)	290.73 (38)	279.71 (38)		255.72 (37)	245.80 (37)	
*N*	4,356	4,957		3,192	3,472	

*Notes*: Report odds ratios with standard errors in parentheses from logit models. Covariate state of resident was omitted.

***, **, *, + denote statistical significance at the 0.1, 1, 5, and 10 percent levels, respectively.

## HIGHLIGHTS

- Landownership is an important source of old age security in India.
- Agricultural land in India is typically controlled by the patriarch; women rarely own or control household land.
- The generational power conferred on older men with landownership does not apply to older women to the same degree.
- It is crucial to register household land under women’s names and recognize women as actual landowners.

## APPENDIX

**Table A1:** Older people’s landownership and household farm work participation by gender and control over farm-related decisions in the IHDS-II

	Primary decision maker of farm		Not primary decision maker of farm	
	Older men	Older women	Older men	Older women
Joint owner of land (%)	10.9	20.4	14.7	5.6
Sole owner of land (%)	76.5	47.9	43.7	5.5
Work in household farm (%)	96.1	93.0	27.2	24.4

**Table A2:** Weighted means (standard deviations) or percentage distributions for selected covariates by older people’s gender and landownership in the IHDS-II

	Older men		Older women	
	Landed household	Landless household	Landed household	Landless household
Household members’ highest years of education	9.23 (4.49)	7.89 (4.49)	8.77 (4.56)	7.57 (4.72)
Household caste and religion (%)				
Forward	26.2	12.0	26.2	14.4
OBC	42.9	33.2	42.2	33.6
Dalit	14.1	33.8	15.6	32.3
Adivasi	7.6	6.0	7.5	6.1
Muslim	7.4	13.5	6.5	11.3
Christian/Sikh/Jain	1.9	1.6	2.0	2.4
Mean household income	133,544.7 (183,398.3)	102,976.3 (93,695.21)	125,898.2 (185,396.3)	100,453.8 (124,558.0)
No. of household assets	13.99 (5.56)	13.44 (5.39)	13.82 (5.56)	13.56 (5.59)
Owned house (%)	99.8	98.4	98.7	96.7
Land size (hectares)	1.45 (2.05)	0	1.46 (2.37)	0
	Landowner	Not landowner	Landowner	Not landowner
Household members’ highest years of education	9.11 (4.57)	9.60 (4.04)	9.23 (4.48)	8.70 (4.54)
Household caste and religion (%)				
Forward	25.8	27.4	26.8	26.1
OBC	43.1	42.2	38.7	42.8
Dalit	13.3	16.5	16.4	15.5
Adivasi	7.9	6.4	11.6	6.8
Muslim	7.8	5.9	3.4	7.0
Christian/Sikh/Jain	2.1	1.5	3.2	1.8
Mean household income	134,447.9 (176,324.7)	130,597.2 (198,165.7)	134,231.9 (237,299.2)	124,524.4 (174,600.7)
No. of household assets	13.85 (5.53)	14.46 (5.43)	14.55 (5.63)	13.70 (5.51)
Owned house (%)	99.8	99.7	99.4	98.6
Land size (hectares)	1.46 (2.06)	1.44 (1.93)	1.94 (2.59)	1.38 (2.31)
	Sole landowner	Not sole landowner	Sole landowner	Not sole landowner
Household members’ highest years of education	8.90 (4.64)	9.82 (4.05)	8.57 (4.63)	8.79 (4.53)
Household caste and religion (%)				
Forward	24.8	28.7	22.4	26.5
OBC	43.6	41.6	38.1	42.6
Dalit	14.0	14.2	21.5	15.1
Adivasi	8.4	6.1	11.9	7.1
Muslim	7.5	7.2	3.3	6.8
Christian/Sikh/Jain	1.8	2.3	2.7	2.0
Mean household income	123,869.0 (155,515.8)	150,915.2 (220,036.9)	123,409.5 (267,604.3)	126,108.2 (176,291.6)
No. of household assets	13.49 (5.43)	14.90 (5.54)	13.77 (5.91)	13.83 (5.51)
Owned house (%)	99.9	99.7	99.0	98.7
Land size (hectares)	1.20 (1.59)	1.90 (2.59)	1.08 (1.24)	1.49 (2.42)
	Decision maker	Not decision maker	Decision maker	Not decision maker
Household members’ highest years of education	9.46 (4.40)	8.84 (4.52)	9.20 (3.83)	8.75 (4.57)
Household caste and religion (%)				
Forward	26.5	25.6	26.9	26.1
OBC	41.1	45.8	29.8	42.9
Dalit	13.6	14.8	24.8	15.1
Adivasi	8.0	6.8	11.6	7.3
Muslim	8.6	5.3	4.1	6.6
Christian/Sikh/Jain	2.1	1.7	2.9	2.0
Mean household income	136,580.2 (178,044.0)	128,483.4 (187,884.8)	152,958.9 (298,471.8)	124,332.0 (175,704.6)
No. of household assets	14.08 (5.49)	13.85 (5.54)	13.95 (5.76)	13.82 (5.53)
Owned house (%)	99.8	99.9	89.1	99.3
Land size (hectares)	1.38 (1.81)	1.57 (2.35)	1.20 (1.66)	1.47 (2.39)

**Table A3:** Weighted means (standard deviations) or percentage distributions for selected covariates by gender and landownership in the IHDS-I

	Older men		Older women	
	Landed household	Landless household	Landed household	Landless household
Household caste and religion (%)				
Forward	27.0	12.7	27.5	13.0
OBC	42.0	32.4	39.5	3.5
Dalit	13.7	35.1	14.7	32.0
Adivasi	6.7	5.5	8.3	6.0
Muslim	7.7	11.2	6.9	9.5
Christian/Sikh/Jain	2.9	3.1	3.1	4.6
Mean household income	115,040.10 (198,437.60)	78,051.16 (91,162.23)	107,199.30 (187,676.30)	68,385.83 (73,789.05)
No. of household assets	11.27 (5.17)	9.92 (4.98)	11.01 (5.22)	9.76 (4.99)
Owned house (%)	99.8	98.2	99.7	96.7
Land size (hectares)	1.99 (3.33)	0	1.97 (0.09)	0
	Decision maker	Not decision maker	Decision maker	Not decision maker
Household caste and religion (%)				
Forward	26.4	28.1	22.8	27.9
OBC	41.9	41.8	42.9	39.3
Dalit	13.8	13.6	14.0	14.6
Adivasi	6.7	6.8	7.0	8.3
Muslim	8.4	6.6	8.9	6.8
Christian/Sikh/Jain	2.8	3.1	4.5	3.1
Mean household income	116,101.10 (216,194.80)	113,474.82 (169,058.90)	106,761.90 (220,570.10)	107,517.50 (184,202.30)
No. of household assets	11.16 (5.23)	11.42 (5.04)	10.87 (5.89)	11.04 (5.12)
Owned house (%)	99.8	99.7	99.7	99.7
Land size (hectares)	2.02 (3.66)	1.95 (2.77)	1.04 (1.90)	2.02 (3.29)

**Table A4:** Odds ratios for binary logit models predicting older men and women dying between the IHDS-I and IHDS-II by landownership in the IHDS-II (sample including older people not living in multi-generational households)

	Household owns land (ref. no)			Decision-maker (ref. no)		
	Model 1a Men	Model 1b Women	M vs. W	Model 2a Men	Model 2b Women	M vs. W
Yes	0.827+ (0.083)	1.046 (0.106)	*p* = 0.100	0.469*** (0.053)	0.563** (0.123)	*p* = 0.452
Age	1.255* (0.129)	1.060 (0.088)		1.280* (0.147)	1.151 (0.115)	
Age squared	0.999 (0.001)	1.000 (0.001)		0.999 (0.001)	1.000 (0.001)	
Widowed/divorced (ref. married)	1.228*** (0.071)	1.276*** (0.072)		1.192* (0.084)	1.325*** (0.085)	
Years of education	0.960** (0.014)	0.911*** (0.024)		0.971 (0.018)	0.907* (0.035)	
Household caste and religion (ref. Forward)						
OBC	0.812+ (0.101)	1.073 (0.137)		0.871 (0.128)	1.209 (0.183)	
Dalit	0.952 (0.130)	1.123 (0.160)		1.065 (0.179)	1.082 (0.218)	
Adivasi	1.075 (0.179)	1.781** (0.368)		1.036 (0.201)	1.854* (0.451)	
Muslim	0.953 (0.184)	1.248 (0.225)		1.100 (0.256)	1.162 (0.280)	
Christian/Sikh/Jain	0.981 (0.236)	1.167 (0.316)		0.915 (0.395)	0.940 (0.338)	
Household income (logged)	0.881* (0.051)	0.940 (0.048)		0.912 (0.056)	0.957 (0.060)	
No. of household assets	0.975* (0.012)	0.985 (0.011)		0.974+ (0.015)	0.981 (0.013)	
Land size (hectares)	1.000 (0.000)	1.000 (0.000)		1.000 (0.000)	1.000 (0.000)	
Owned house (ref. not owned)	0.725 (0.281)	0.566 (0.201)		0.324 (0.264)	0.475 (0.309)	
No. of household adult men	0.959 (0.062)	1.003 (0.060)		0.817** (0.060)	1.040 (0.073)	
No. of household adult women	1.158* (0.083)	1.132+ (0.081)		1.158+ (0.091)	0.970 (0.080)	
Wald (df)	282.46 (38)	299.84 (38)		215.23 (37)	254.45 (37)	
*N*	5,940	5,912		4,116	3,978	

*Notes*: Report odds ratios with standard errors in parentheses from logit models. Independent variable state of resident was omitted.

***, **, *, + denote statistical significance at the 0.1, 1, 5, and 10 percent levels, respectively.
